# Effect of a bioconverted product of *Lotus corniculatus* seed on the axillary microbiome and body odor

**DOI:** 10.1038/s41598-021-89606-5

**Published:** 2021-05-12

**Authors:** Min-Ji Kim, Setu Bazie Tagele, HyungWoo Jo, Min-Chul Kim, YeonGyun Jung, Yeong-Jun Park, Jai-Hyun So, Hae Jin Kim, Ho Jin Kim, Dong-Geol Lee, Seunghyun Kang, Jae-Ho Shin

**Affiliations:** 1 Department of Applied Biosciences, Kyungpook National University, Daegu, 41566 Republic of Korea; 2 R&I Center, COSMAX BTI, Seongnam, 13486 Republic of Korea; 3 National Development Institute of Korean Medicine, 94, Hwarang-ro, Gyeongsan, Gyeongsangbuk-do 38540 Republic of Korea; 4 Experiment Research Institute, National Agricultural Products Quality Management Service, Gimcheon, Gyeongsangbuk-do 39660 Republic of Korea

**Keywords:** Microbiology, Applied microbiology

## Abstract

The skin microbiome, especially the axillary microbiome, consists of odor-causing bacteria that decompose odorless sweat into malodor compounds, which contributes to the formation of body odor. Plant-derived products are a cheap source of bioactive compounds that are common ingredients in cosmetics. Microbial bioconversion of natural products is an ecofriendly and economical method for production of new or improved biologically active compounds. Therefore, in this study, we tested the potential of a *Lactobacillus acidophilus* KNU-02-mediated bioconverted product (BLC) of *Lotus corniculatus* seed to reduce axillary malodor and its effect on the associated axillary microbiota. A chemical profile analysis revealed that benzoic acid was the most abundant chemical compound in BLC, which increased following bioconversion. Moreover, BLC treatment was found to reduce the intensity of axillary malodor. We tested the axillary microbiome of 18 study participants, divided equally into BLC and placebo groups, and revealed through 16S rRNA gene sequencing that *Staphylococcus*, *Corynebacterium,* and *Anaerococcus* were the dominant taxa, and some of these taxa were significantly associated with axillary malodor. After one week of BLC treatment, the abundance of *Corynebacterium* and *Anaerococcus*, which are associated with well-known odor-related genes that produce volatile fatty acids, had significantly reduced. Likewise, the identified odor-related genes decreased after the application of BLC. BLC treatment enhanced the richness and network density of the axillary microbial community. The placebo group, on the other hand, showed no difference in the microbial richness, odor associated taxa, and predicted functional genes after a week. The results demonstrated that BLC has the potential to reduce the axillary malodor and the associated odor-causing bacteria, which makes BLC a viable deodorant material in cosmetic products.

## Introduction

Human body odor generally creates an adverse impression in human-to-human interaction, causing psychological stress and negatively impacting social life in afflicted humans. Personality attributes, facial attractiveness, and health status are profoundly influenced by body odor^1–3^. The skin microbiome, particularly the armpit region, is covered by dense secretory glands, such as apocrine, eccrine, and sebaceous glands. These glands secret various nutrients and moisten skin folds such as the armpit, which provides habitable growth conditions for odor-causing bacteria^4^. The bacterial community in the armpit decomposes the odorless sweat into volatile odorous byproducts, such as volatile fatty acids (VFAs), 3-hydroxy hexanoic acid (3M3H), and 3-hydroxy-3-methylhexanoic acid (HMHA)^5^. Thus, the axillary microbiome plays a key role in the generation of malodor, making it a target for the inhibition of odor-producing bacteria as a means to substantially reduce body odor.

As body odor negatively affects personal life, numerous products that reduce malodor have been developed, including fragrances, antiperspirants, and deodorants, which mask body odor, suppress sweat secretion, and/or inhibit bacterial growth. However, these products carry their own risks upon exposure^6–9^. Masking body odor with a pleasant scent exacerbates odor-producing conditions due to a combination of fragrance and sweat, and exposure to certain ingredients in fragrances can cause allergic reactions^6^. In addition, aluminum salt is found in many antiperspirants to inhibit sweating, despite being a risk factor for Alzheimer’s disease and accelerating inflammatory reactions^7,8^. Deodorants with added fragrances can cause allergic reactions^9^. Therefore, it is necessary to find a substance that can reduce body odor without any adverse effects.

Natural ingredients, especially plant-derived products, have long been used in traditional medicine^10,11^ and are a cheap source of bioactive compounds that control antibiotic resistance^12^. Plant extracts have antioxidant, anti-inflammatory, and anticancer activities^13^. Phenolic compounds from plant extracts, which have antimicrobial and antiradical activities, have long been used as skin care ingredients^14^. The potential of phytochemicals to treat skin diseases has been previously documented^15^. Plant extracts, which are rich in piceatannol, are good skin care materials, as they moisturize dry skin^16^. Likewise, plant materials, which are also used as fragrance and deodorant substances for cosmetics, soaps, and perfumes, are also used to reduce odor, as they retain floral fragrances and absorb odor^17^. In addition, plants such as sage and tea leaves have been used as odor-reducing substances because of their antimicrobial activity^18,19^. *Lotus corniculatus,* which is an acidic-tolerant flowering plant containing condensed tannins^20^, has been found to show promising antimicrobial and anticancer properties^21–23^. Furthermore, *L. corniculatus* has been reported to possess substantial healing effects on skin wounds^24^. However, the effect of *L. corniculatus* extract on axillary odor and the associated microbiota has not been reported yet*.*

A bioconversion technique, which is the process of converting organic materials into usable products by biological agents, is an economically and ecologically important practical technology that is widely used to modify the structure of many types of biologically active products^25^. In addition, novel products can be isolated with new or different activities, such as antimicrobial and antioxidant properties^25–27^. In a previous study, probiotic bacterial bioconversion increased the antiradical activity of polyphenols in lotus seed epicarp^28^. Lactic acid bacteria (LAB) has been widely used for the bioconversion of natural products and in the food industry to ferment beverages^29,30^.

In this study, we bioconverted seeds of *L. corniculatus* using a LAB isolate, *Lactobacillus acidophilus* KNU-02. Next, we evaluated the effect of the bioconverted product of *L. acidophilus* KNU-02 (BLC) on the axillary microbiota structure and body odor. To validate the efficacy of BLC, we measured the odor intensity and collected armpit swab samples (for 16S rRNA gene sequencing) from 18 female Korean volunteers at baseline (0 week) and after one week of BLC treatment (1 week). Our study demonstrated that BLC reduced axillary malodor and inhibited odor-causing bacteria. This implies that BLC could be a potential source of ingredients for cosmetics.

## Results

### Bioconversion altered chemical profile of *L. corniculatus* seed extract

**Figure 1:**
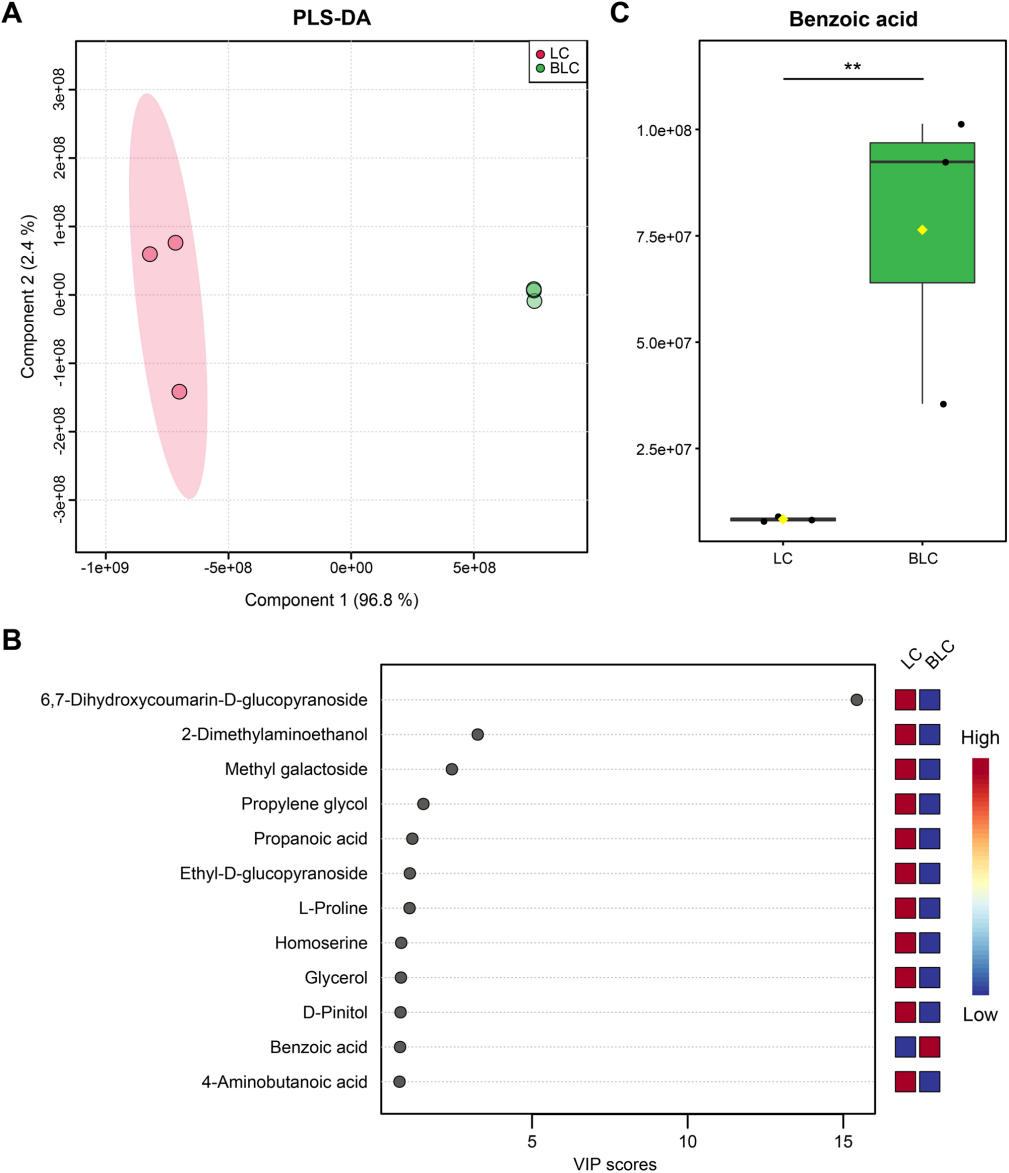
Compositional analysis of bioconverted product (BLC) and the seed extract (LC). (**A**) PLS-DA score plot using all features. (**B**) PLS-DA variable importance in projection (VIP) score plot. The features were selected with a VIP score > 0.7 based on the corresponding PLS-DA model. (**C**) Box plot of normalized peak abundances for benzoic acid.

To identify changes in the chemical profile of the BLC seed extract, we analyzed the chemical compositions of BLC and the original seed extract using GCxGC/TOF–MS. The chemical profile of BLC showed that 12 major chemical compounds were more than 1% of the content. The predominant metabolites, each formed more than 3% of the profile, were benzoic acid, silanol, palmitic acid, propylene glycol, 4-hydroxybenzoic acid, and D-lactic acid (Table 1). Among the compounds, benzoic acid was the most abundant (17.9%) in BLC, followed by silanol and palmitic acid metabolites (14.6%). Note that benzoic acid was one of the least (< 1%) abundant in the original seed extract (Supplementary Table S1). The results of the partial least squares discriminant analysis (PLS-DA) revealed that the chemical composition of the BLC and original seed extract (LC) were distinct from each other. The total detected variation was 96.8% component1 and 2.4% component2 in PLS-DA (Fig. 1A). In addition, variable importance in projection (VIP) scores were used to identify discriminatory chemical compounds during the bioconversion process. 12 chemical compounds were identified as key features to discriminate BLC from LC (VIP > 0.7) (Fig. 1B, Supplementary Table S2). Among the key features that discriminate between the two extracts, only benzoic acid, a well-known cosmetic ingredient, was markedly increased (Fig. 1B,C) following the bioconversion process. Furthermore, the bioconversion process also resulted in a decline in the abundance of propionic acid (Fig. 1B), which can be detected by the strength of its pungent smell, implying the significance of this bioconversion technique.

**Table 1:** The list of major compounds (> 1%) identified in bioconverted product by GCxGC/TOF–MS analysis.

Compound	R.T. (s)	MW (g/mol)	Formula	Area (%)
Benzoic acid, 3,4-bis^[^(trimethylsilyl)oxy^]^-, trimethylsilyl ester (CAS)	786.23	370.66	C_16_H_30_O_4_Si_3_	17.90
Silanol, trimethyl-, phosphate (3:1) (CAS)	483.46	314.54	C_9_H_27_O_4_PSi_3_	8.143
Palmitic Acid, TMS derivative	936.61	328.61	C_19_H_40_O_2_Si	6.532
Propylene glycol, 2TMS derivative	304.70	220.46	C_9_H_24_O_2_Si_2_	5.045
4-Hydroxybenzoic acid, 2TMS derivative	671.69	282.48	C_13_H_22_O_3_Si_2_	3.517
D-Lactic acid-DITMS	346.62	234.44	C_9_H_22_O_3_Si_2_	3.307
Propanoic acid, 3-^[^(trimethylsilyl)oxy^]^-, trimethylsilyl ester (CAS)	400.76	234.44	C_9_H_22_O_3_Si_2_	2.853
D-(-)-Ribofuranose, tetrakis(trimethylsilyl) ether (isomer 2)	639.13	438.85	C_17_H_42_O_5_Si_4_	1.449
D-Psicose, pentakis(trimethylsilyl) ether, methyloxime (anti)	828.64	570.10	C_22_H_55_NO_6_Si_5_	1.265
Maltol, TMS derivative	494.95	198.29	C_9_H_14_O_3_Si	1.161
D-Fucitol, 5TMS derivative	764.81	527.08	C_21_H_54_O_5_Si_5_	1.136
6,7-Dihydroxycoumarin-á-D-glucopyranoside, PENTA-TMS	1470.8	700.00	C_30_H_56_O_9_Si_5_	1.056

### Effect of the bioconverted product on body odor intensity

**Figure 2:**
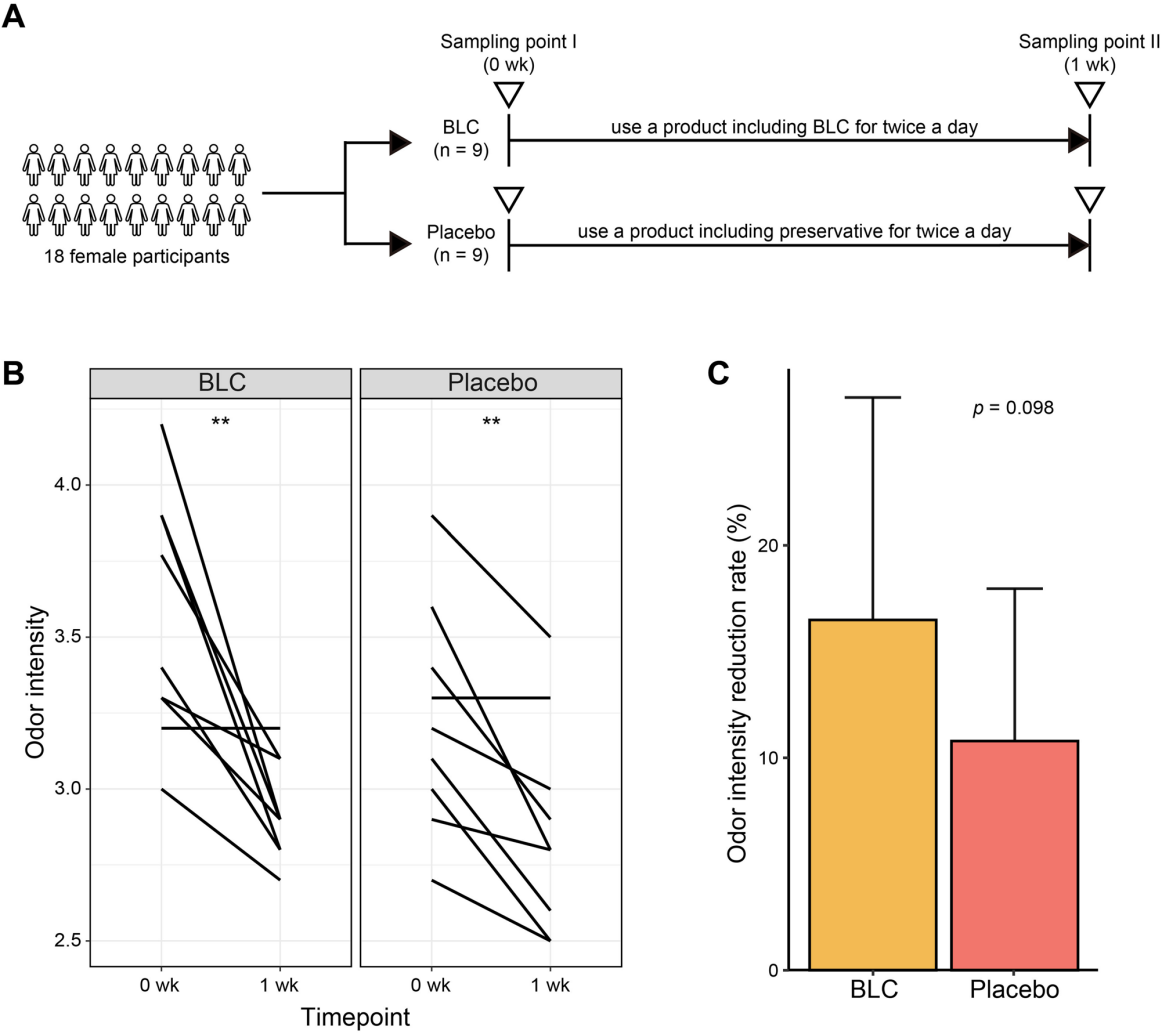
The study design and axillary odor intensity. (**A**) Pictorial representation of the study design. (**B**) The axillary odor intensity changes in each group. (**C**) Reduction rate of odor intensity in each group. A Wilcoxon matched-pairs test was conducted to compare the odor intensity between 0 and 1 week. The comparison of the odor intensity reduction rate between the two groups was evaluated with an unpaired t-test. A *p* value  < 0.01 is indicated with asterisks (**).

In this study, we determined the potential of BLC to reduce axillary malodor. We recruited nine participants for each treatment group (BLC and placebo groups). The two groups had no significant axillary odor intensity, age and gender differences at the baseline (0 week) (Supplementary Table S3). However, a week after treatment (1 week), odor intensity was significantly reduced in both groups; BLC had a significantly (*p* < 0.1) higher odor intensity decline than the placebo (Fig. 2B,C). This signifies the effectiveness of the BLC treatment in reducing body malodor.

### Bioconverted product altered axillary microbiome

**Figure 3:**
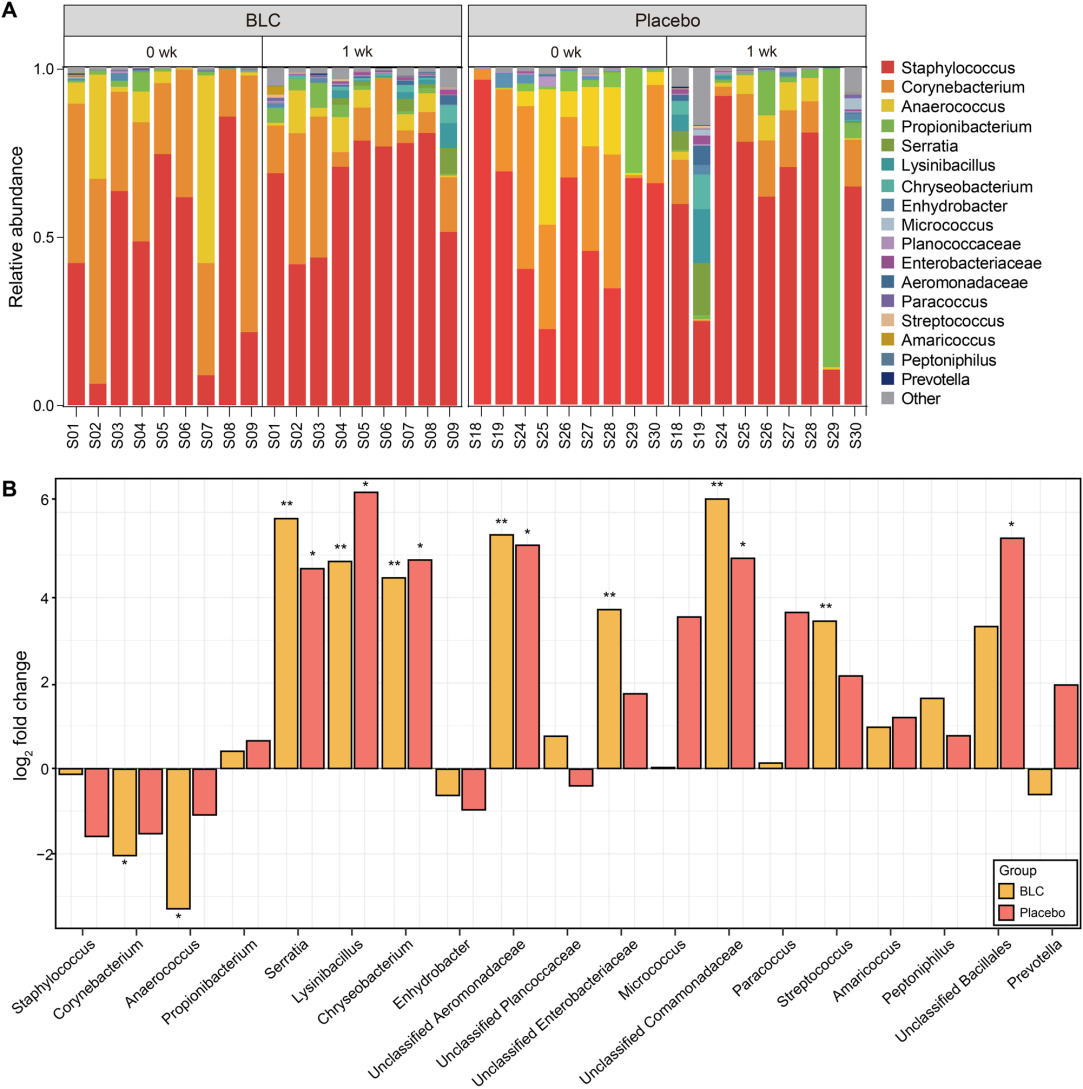
Taxonomic changes in the axillary microbiome for bioconverted product (BLC) and placebo. (**A**) The relative abundance of the axillary microbiome at the genus level. (**B**) Log2-fold-change in the BLC (yellow) and placebo (red), which represents changes at 1 week after using applications in comparison with those of the baseline samples. False discover rate-adjusted values < 0.05 and 0.01 are indicated with asterisks (* and **, respectively).

We also investigated the effect of BLC on the axillary microbiome of the study participants. Armpit swab samples were collected at 0 week and 1 week. Our results showed that *Staphylococcus, Corynebacterium*, *Anaerococcus*, and *Propionibacterium* were the predominant genera in the axillary microbiome of the two groups at 0 week and 1 week (Fig. 3A). The taxonomic differences between 0 and 1 week in BLC and placebo groups were compared, and the top 19 abundant taxa were included in the test. Nine and six taxa were found to be differentially abundant taxa at 1 week compared with that at 0 week in the BLC and placebo groups, respectively (Fig. 3B). Among the three dominant taxa, viz., *Staphylococcus*, *Corynebacterium,* and *Anaerococcus,* the latter two were significantly decreased (false discover rate [FDR] < 0.05) at 1 week following BLC treatment compared to those at 0 week. However, these two genera did not show significant differences from the placebo group after a week. Furthermore, seven taxa, including *Streptococcus*, Enterobacteriaceae, *Chryseobacterium*, *Lysinibacillus*, Aeromonadaceae, *Serratia,* and Comamonadaceae, were enriched at 1 week following BLC treatment. In the placebo at 1 week, six taxa, Aeromonadaceae, Bacillales, *Chryseobacterium*, Comamonadaceae, *Lysinibacillus*, and *Serratia,* were significantly (FDR > 0.05) increased compared with those at 0 week.

**Figure 4:**
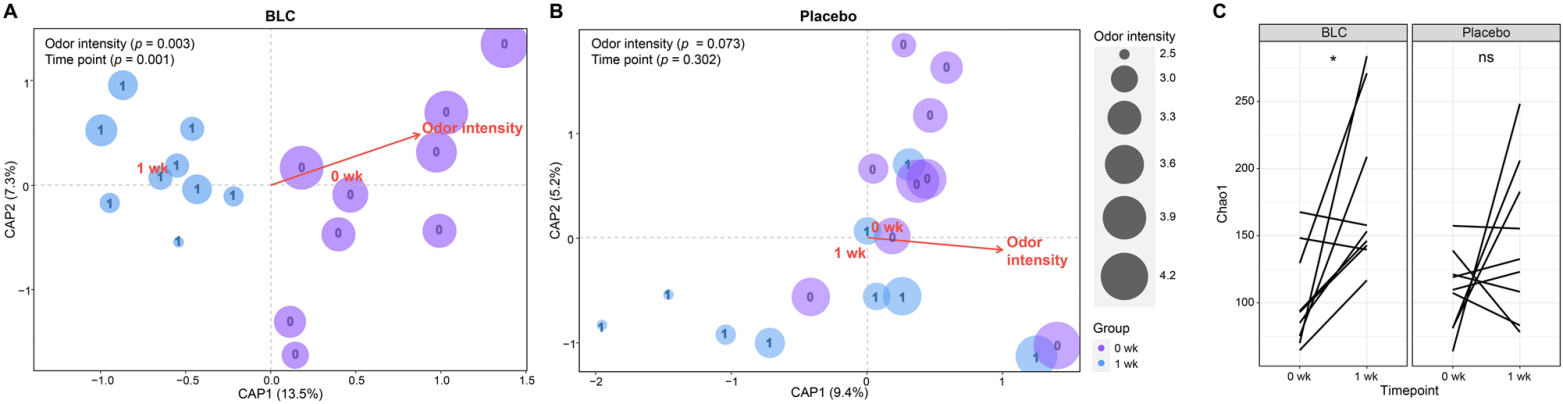
Shifts in the axillary microbiome of the bioconverted product (BLC) and placebo. Canonical analysis of the principal coordinates of the axillary microbiome in relation to the odor intensity and time point in the (**A**) BLC and (**B**) placebo groups. (**C**) The Chao1 index of each group. Paired t-test used to compare the Chao1 index. The color code and size of the canonical analysis of principal coordination analysis are the group and odor intensity, respectively.

Canonical analysis of principal coordination (CAP) was based on the unweighted UniFrac distance metric. According to the CAP analysis, the BLC group showed a significantly different microbiota structure at 1 week compared with that at 0 week (*p* = 0.001), and there was a strong association with odor intensity (*p* = 0.003) (Fig. 4A). However, as expected, the placebo showed no significant difference between 0 and 1 week (*p* = 0.302) (Fig. 4B). In addition, there was no association between odor intensity and the axillary microbiome in the placebo (*p* = 0.073) (Fig. 4B). Furthermore, the placebo exhibited no changes between 0 and 1 week in all alpha diversity indices (Fig. 4C and Supplementary Fig. S1). On the contrary, BLC showed a significant increase of operational taxonomic unit (OTU) richness, as indicated by the Chao1 index (*p* = 0.014) (Fig. 4C), but not in the remaining alpha diversity indices (Supplementary Fig. S1).

We further explored the association between the top 4 genera and body odor using Spearman’s rank correlation coefficient (Table 2). In the correlation analysis, *Corynebacterium* (*ρ* = 0.422, *p* value = 0.010) and *Anaerococcus* (*ρ* = 0.416, *p* value = 0.012) were significantly positively correlated with odor intensity. Additionally, to adjust the effect of other taxa to one taxon*,* the association between the top 4 genera and odor intensity was further explored using multiple regression model. Among 4 genera, only *Corynebacterium* and *Anaerococcus* had significant association with odor intensity (Supplementary Figure S2). Collectively, our results showed that the BLC treatment reduced body odor by increasing the microbial diversity and inhibiting the growth of odor-causing bacteria, viz., *Corynebacterium* and *Anaerococcus,* in the axillary microbial community.

**Table 2:** Correlation of the abundant 4 genera with odor intensity.

Genus	Spearman’s rho	
	*ρ*	*p* value
*Staphylococcus*	− 0.183	0.285
*Corynebacterium*	0.422	0.010
*Anaerococcus*	0.416	0.012
*Propionibacterium*	− 0.187	0.275

### Network analysis

**Figure 5:**
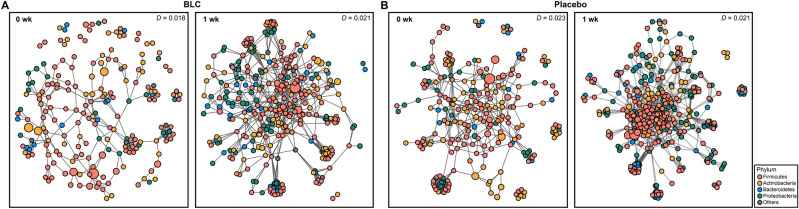
Network analysis of the axillary microbiome at the bacterial operational taxonomic unit (OTU) level. (**A**) Network analyses of the bioconverted product treatment at 0 week and 1 week. (**B**) Network analyses of the placebo at 0 week and 1 week. Color markings indicate the phylum. Each node represents the OTUs, and the size of each node corresponds to the relative abundance. The *D* values, which represent the density, were calculated using the ratio of the number of edges.

To investigate the interaction of OTUs in the axillary microbiome, OTU networks for each group (BLC and placebo) at 0 week and 1 week were generated by Sparse InversE Covariance estimation for Ecological Association Inference (SPIEC-EASI). The OTUs with < 0.01% relative abundance were not included in the analysis. Therefore, the network for the BLC treatment had 203 and 322 nodes at 0 week and 1wk, respectively. In the placebo, 228 and 362 nodes were obtained at 0 week and 1 week, respectively. The network density (*D*) of the axillary microbiome was calculated using the ratio of the number of edges for each time point in each group. The network analysis demonstrated that the *D* value was slightly higher following BLC treatment after 1 week (*D* = 0.021) than at to 0 week (*D* = 0.018) (Fig. 5A). On the other hand, the *D* value slightly decreased in the placebo after 1 week (*D* = 0.021) than at 0 week (*D* = 0.023) (Fig. 5B). This could be due to the reduced dominance of odor-causing taxa and the subsequent increase in the abundance of other bacterial taxa in the axillary microbiota after one week. Furthermore, the network topological coefficients were evaluated for each group (Supplementary Table S4). The network transitivity (*T*) value increased after one week compared to 0 week. The number of edge, degree, and node closeness centrality of the network were also increased after two treatments. The higher the degree, the higher the node dependence. Additionally, an increase of the node closeness centrality implies that the microbial network is the hub-based network which has high connection with other nodes. These results indicate that the BLC increased interdependence among bacteria in the community. Therefore, BLC makes the network more compact, complex and stable.

### Functional gene prediction using PICRUSt

**Figure 6:**
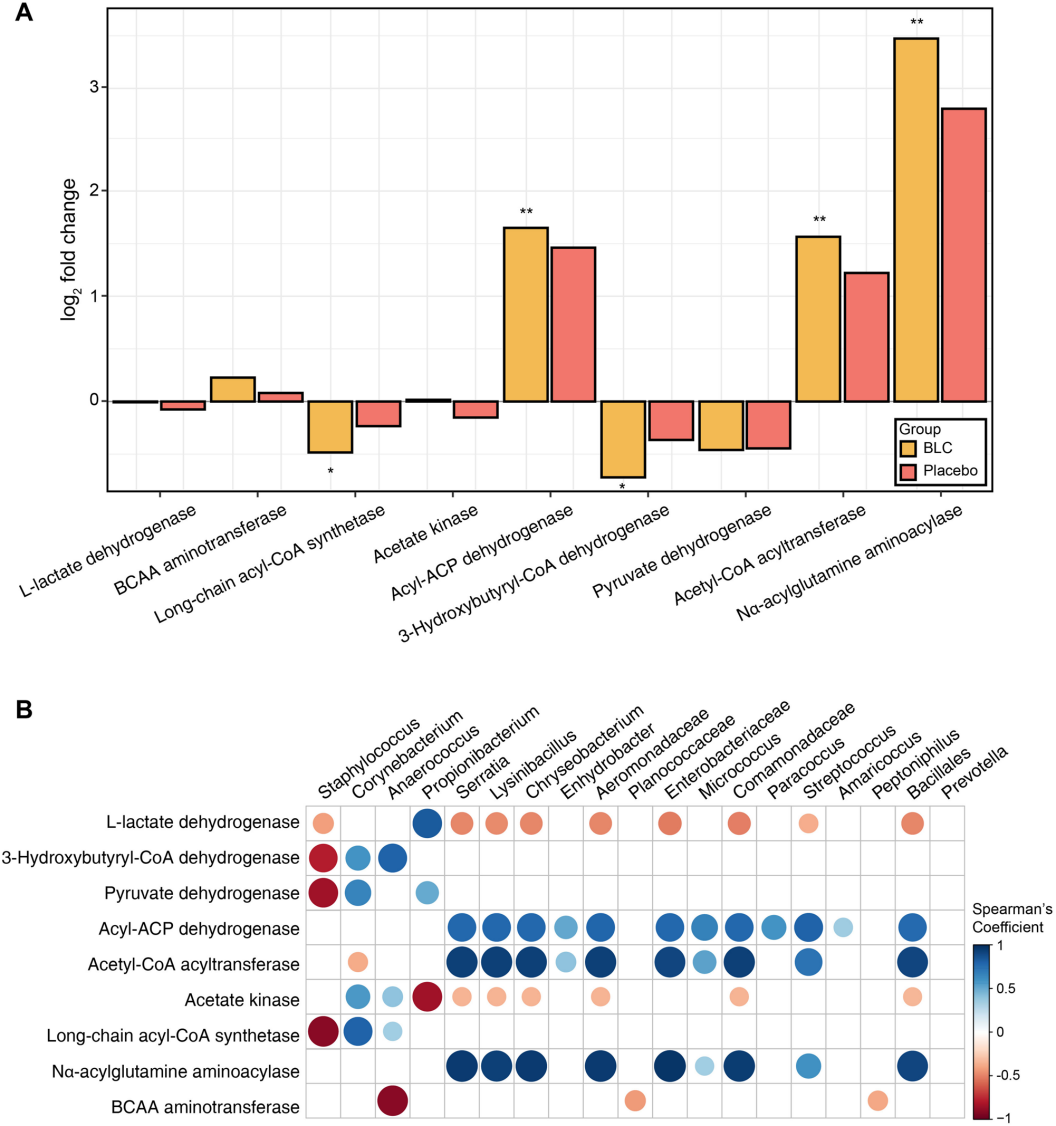
Functional gene prediction analysis of the axillary microbiome. (**A**) Log2-fold-change of odor-related genes in the bioconverted product (yellow) and placebo (red) group that represented changes at 1 week after using products in comparison with the baseline samples. False discover rate-adjusted values less than 0.05 and 0.01 are indicated with asterisks (* and **, respectively). (**B**) Correlation matrix showing a significant correlation (*p* < 0.05) between the most abundant taxa and odor-related genes. The color code and size of the circles are the *ρ* correlation coefficient.

To gain insight into the metabolic function of the microbial community in the armpit of the participants, functional gene prediction of the axillary microbiome from 16S rRNA gene sequencing was conducted using Phylogenetic Investigation of Communities by Reconstruction of Unobserved States (PICRUSt). Functional genes that were predicted to be related to the production of odorous compounds were analyzed. Among the odor-related genes, five genes that were predicted to be involved in odor production exhibited significant (FDR < 0.05) differential expression at 1 week of BLC treatment compared with that at 0 week (Fig. 6A). However, in the placebo, there were no significantly differentially expressed genes at 1 week compared with the baseline. After one week of BLC treatment, the two predicted genes that showed a significant decrease in abundance were found to encode long-chain acyl-CoA synthetase (FadD) and 3-hydroxybutyryl-CoA dehydrogenase (FadB). Long-chain acyl-CoA synthetase and 3-hydroxybutyryl-CoA dehydrogenase are essential enzymes for the production of VFAs by catabolizing lipids during β-oxidation cycle. In contrast, the remaining three genes that showed an increment in abundance were acyl-ACP dehydrogenase, acetyl-CoA acyltransferase, and N^α^-acylglutamine aminoacylase. However, the relative abundance of these three genes in the axillary microbiome was less abundant, which might reduce their impact on body odor (Supplementary Table S5). Indeed, the total sum of the relative abundance of the predicted genes with odor-related functions decreased at 1 week of BLC treatment compared with that at 0 week (Supplementary Table S5). This implies that the BLC treatment significantly affected the odor-related functions of the axillary microbiota, as predicted.

Further correlation analysis was performed to determine which taxa had contributed more to the change in functional genes. Hence, we analyzed the correlation between the most abundant taxa and odor-related functional genes using Spearman’s rank correlation coefficient (Fig. 6B). Interestingly, at the genus level, *Corynebacterium* had a positive correlation with the four functional genes involved in odor production, namely 3-hydroxybutyryl-CoA dehydrogenase, pyruvate dehydrogenase, acetate kinase, and long-chain acyl-CoA synthetase. Moreover, *Anaerococcus* was also related to the four genes encoding 3-hydroxybutyryl-CoA dehydrogenase, acetate kinase, long-chain acyl-CoA synthetase and branched-chain amino acid (BCAA) aminotransferase. *Anaerococcus* had a positive correlation with all the above-mentioned genes except BCAA aminotransferase. *Corynebacterium* and *Anaerococcus* were positively associated with body-odor-related genes, such as 3-hydroxybutyryl-CoA dehydrogenase, pyruvate dehydrogenase, acetate kinase, and long-chain acyl-CoA synthetase. Furthermore, the contribution of each taxa to odor-related genes was also performed using metagenome_contributions.py command line in PICRUSt algorithm. *Staphylococcus, Corynebacterium* and *Anaerococcus* contributed the most to odor-related genes (Supplementary Figure S3). Therefore, the use of BLC plays a key role in reducing odor-related genes, primarily by inhibiting the growth of *Corynebacterium* and *Anaerococcus* in the axillary microbiome.

## Discussion

Microbial bioconversion of natural products has been used to produce new or improved products^31,32^. Such changes in the chemical constituents of bioconverted products are attributed to various chemical reactions, such as methylation, degradation, and glycosylation^33–35^. Several new bioactive compounds in bioconverted products are used for nutrition and health benefits, including anti-allergic, antioxidant, and anticancer effects^32,36^. Furthermore, the use of plant-based products in cosmetic markets is increasing^33,37^. This is due to the fact that plant-based skin care products are cheap and have huge potential to replace synthetic antimicrobials in skin care products^38,39^. These products help body’s cells to repair and reduce signs of skin aging. This has led to the growing of plant-based cosmetic brands and green-beauty market. Nevertheless, the untapped resource of plants for cosmetic purposes, however, has not been well exploited commercially^40^. Therefore, in this study, we determined the potential use of the BLC in reducing axillary odor. We also investigated the effects of BLC treatment on the associated axillary microbiome. At the genus level, the axillary microbiome of the participants mainly consisted of *Staphylococcus*, *Corynebacterium*, *Anaerococcus*, and *Propionibacterium* (Fig. 3A). Our study results support previous reports that established these four gram-positive bacteria as inhabitants of human skin^41,42^. Indeed, these genera are also the major taxa in the axillary region^41–44^. *Staphylococcus* and *Corynebacterium* are dominant taxa in moist body regions, such as the armpit and navel^42,45^. Previous studies demonstrated that *Propionibacterium* is particularly adapted to anaerobic or lipid-rich environments, such as sebaceous glands^46,47^.

We evaluated whether BLC could reduce axillary malodor and alter the axillary microbiome of the study participants. Indeed, the BLC treatment reduced body odor and shifted the axillary microbiome. Although body odor for the two groups decreased, the percentage of odor reduction following BLC treatment was higher than the placebo group (Fig. 2C). The decrease in body odor observed in a placebo group may be due to the placebo effect, which is commonly found in many clinical trials^48,49^. According to the previous studies, the ritual of taking medication^50^ and response expectancy^51^ is the key driving forces in the healing process as the placebo effect can modulate the nervous system-immune system communication^52^. In a separate study, the potential of plant-based extracts to reduce body odor was reported^53^. However, to the best of our knowledge, this is the first report showing the positive effects of a bioconverted product from *L. corniculatus* seed in reducing axillary malodor.

Such difference in odor reduction between the two groups can be explained by the observed difference in the axillary microbiome. Our study showed that the richness of the axillary microbiome increased after BLC treatment (Fig. 4C). Indeed, previous studies also showed that skin care products induce an increase in skin microbial richness^54–56^. The increase in richness may be caused by the colonization of non-skin commensal bacteria. Unlike the placebo, the relative abundance of *Corynebacterium* and *Anaerococcus* significantly decreased due to the BLC treatment (Fig. 3B)*.* According to previous reports, skin commensal bacteria metabolize odorless sweat into volatile odorants^57–59^. *Corynebacterium* is one of the dominant genera that produces various odorants, including VFAs, HMHA, 3M2H, and 5α-androst-16-en-3α-ol^5,60–62^. *Anaerococcus* also plays a role in the production of odorants such as 3-hydroxy-3-methylhexanoic (3H3MH) acid and VFAs^33,39,63^. Moreover, *Staphylococcus*, *Propionibacterium*, and *Micrococcus* are also known to generate volatile odorants, viz., 3-methyl-3-sulfanylhexan-1-ol (3M3SH) and VFAs^64–66^. In the network analysis, BLC induced a more stable community for the axillary microbiome, as indicated by higher network density (*D*) and transitivity values (*T*) than the baseline (Fig. 5A and Supplementary Table S4). In previous studies, low network density and transitivity of the skin microbiome was correlated with dandruff skin and a fragile community, highlighting the need to maintain stable bacterial communities for good skin health^67,68^. Additionally, BLC induced a more complex and compact the axillary microbial community, as indicated by higher values of network topological coefficients including the node closeness centrality, edges, and degree (Supplementary Table S4). According to the previous study^69^, greater node closeness centrality and number of the degree are indicators of compact network with many hub-bacteria. Therefore, BLC induced more compact and stable axillary microbial network compared to placebo.

PICRUSt analysis also revealed that among the odor-associated functional groups^5^, the abundance of FadB and FadD significantly decreased in the BLC-treated group after a week. However, there was no significant decrease in the odor-related metabolic functional groups in the placebo group (Fig. 6A). PICRUSt analysis results are consistent with previous studies that demonstrated that FadD is a crucial membrane-associated functional gene that facilitates fatty acid degradation^70^. Likewise, FadB is also required for the degradation of short-chain fatty acids^70^. VFAs can be generated through β-oxidation cycle^57,60^. β-oxidation cycle is related to fatty acid catabolism, as it is the first cycle to break down long fatty acids into shorter fatty acyl-CoA chains^60,71^. In this study, FadB and FadD were positively correlated with *Corynebacterium* (Fig. 6B). *Corynebacterium* metabolites fatty acid into malodor compounds, such as VFAs, through the β-oxidation cycle^60^. In addition, *Corynebacterium* also exhibited a positive correlation with odor-associated functions, viz., pyruvate dehydrogenase and acetate kinase (Fig. 6B). Regarding properties of *Corynebacterium,* several studies reported that it is the primary cause of axillary malodor^71^. In addition, *Anaerococcus* was also positively related with L-lactate dehydrogenase, 3-hydroxybutyryl-CoA dehydrogenase, and acetate kinase (Fig. 6B). L-lactate dehydrogenase is related to the production of odorants, as it converts lactic acid into pyruvate^66,72^. Through oxidative degradation, pyruvate dehydrogenase converts pyruvate into acetic acid and sour odor^66,73^. Nevertheless, three genes significantly increased in the BLC (Fig. 6A). These genes have no association with abundant bacteria*.* Among the increased genes, acyl-ACP dehydrogenase (FadE) and acetyl-CoA acyltransferase (FadA) degrade fatty acid through the β-oxidation cycle^70^. Although these genes increased with BLC, they cannot metabolite substances when there is a decrease in FadD, which activates the long-chain fatty acid to transport into the inner membrane. Moreover, the abundance of N^α^-acylglutamine aminoacylase is too low to significantly affect body odor production (Supplementary Table S5). Although previous studies presented a strong association of *Staphylococcus* with body odor^74^, we found that most genes had a negative correlation with *Staphylococcus* (Fig. 6B). Our findings are in agreement with the previous report that showed *Anaerococcus* is the key commensal microbiome^75^. Therefore, our study demonstrated that *Corynebacterium* and *Anaerococcus* contribute more than *Staphylococcus* to the body odor of our participants. Therefore, our results indicate that BLC has the potential to reduce body odor by altering the axillary microbiome.

The chemical composition of BLC was also profiled to determine the changes in the chemical profile of the seed extract after bioconversion. Benzoic acid was the predominant compound in BLC after four days of bioconversion (Table 1). Methyl galactoside was predominant and the abundance of benzoic acid was low (< 1%) in the original seed extract (Supplementary Table S1). In antibacterial activity experiment, BLC had antibacterial activity against *Staphylococcus aureus*, but LC had not (Supplementary Figure S4). The difference of antibacterial activity between BLC and LC may be due to benzoic acid. Indeed, benzoic acid and its derivatives have been previously reported to be nonspecific antibacterial agents that have been used as preservative agents^76,77^. Lipophilic acids, such as benzoic acid, inhibit the active uptake of some amino and oxo acids in *Escherichia coli* and *Bacillus subtilis*^78^. Furthermore, benzoic acid has been reported to possess several cosmetic properties that enhance skin health, like antioxidant and anti-inflammatory activity^14^. The relative composition of 4-hydroxybenzoic acid in the bioconverted seed extract was also abundant in the BLC (Table 1). 4-Hydroxybenzoic acid, which is a phenolic derivate of benzoic acid and also a paraben, is frequently used as a preservative in cosmetics and food industries, as it contains antibacterial and antifungal activities^79,80^. Likewise, palmitic acid, which is also abundant in the BLC, has antibacterial activity^81^. Therefore, such compounds with antibacterial properties in the BLC might have contributed to a reduction in the relative abundance of *Corynebacterium* and *Anaerococcus* in the axillary microbiome, thereby reducing the body odor. Palmitic acid has been used as a skin permeation enhancer^82^. Skin permeation enhancers interact with the constituents of a skin’s outermost layer and increase its permeability^83^. Compounds that have a skin permeability property might enhance the efficacy of the BLC as an axillary odor reducer. In addition, since the BLC contains compounds with various properties, such as antioxidant and anti-aging effects, the BLC could be used as a cosmetic ingredient with multiple beneficial properties for skin health. However, although the most commonly dominant odor-causing bacteria are only few in number^5^, it is worth considering race/ethnicity and gender variations in terms of the axillary microbiome^43^ and skin factors such as skin permeability, sweat secretion and skin hydration level^84^.

In conclusion, to the best of our knowledge, the present study reports for the first time the potential of a microbial bioconverted product of BLC to reduce axillary malodor and alter the associated microbiota. The bioconversion process resulted in the predominance of benzoic acid, which is known to possess several cosmetic application properties that enhance skin health. BLC was found to alter the axillary microbiota and thereby the axillary odor. The BLC treatment was effective in reducing the abundance of the most dominant and odor-associated taxa, *Corynebacterium* and *Anaerococcus*. The BLC treatment also increased the richness and slightly enhanced the network density of the axillary microbial community. Thus, the BLC contains cheap bioactive compounds and could be a potential source of ingredients for cosmetics. Additionally, as *Corynebacterium* and *Anaerococcus* are most common odor-causing bacteria across race/ethnicity and gender group^5,43^, BLC could be an ingredient for universal skin-care products. However, further research with a large number of participants is warranted to ensure the potential of the BLC for future applications in cosmetic products.

## Methods

### Subject recruitment and sample collection

Total 18 Korean women, without chronic skin diseases, were recruited for this study. The participants were divided into two groups, the BLC and the placebo groups. Participants were assigned divided to BLC and placebo groups. Additionally, the treatment assignment was randomized by double-blind test without informing to the experimenters and participants about the distinction between BLC and placebo. Participants of each group were asked to apply their respective treatments twice a day for one week, and they were asked to stop taking showers or using any other products, such as lotion, deodorant, or antiperspirant, on their armpits for at least 12 h prior to the axillary microbiota sample collection (Fig. 2A). Sampling was conducted in a controlled room at 20–24 °C, 45–55% RH. During the 30 min prior to sampling, the participants stayed still without moving their armpit. For the sample collection, a 50 cm^2^ area from the armpit of each subject was swabbed with sterile cotton-tipped swabs pre-moistened with a preservative, which contained 0.1% Tween 20, 0.15 M sodium chloride, and 0.1% agar. The sampling area was swabbed for at least 3 min. Each swab was placed into a tube containing a preservative, immediately frozen, and stored at -80 °C prior to DNA extraction. The odor intensity of the axillae of the participants was assessed at 0 week and 1 week by using an odor meter (Shinyei OMX-ADM, Japan) before axillary microbial collection. In addition, all participants were required to answer a questionnaire related to body odor at 0 week and 1 week. This study was approved by the Institutional Review Boards (IRBs) of the Global Medical Research Center (IRB: GIRB-19O01-AK). Both verbal and written informed consent was obtained from all participants. We confirm that all methods used in the study were performed in accordance with the relevant guidelines and regulations.

### DNA extraction and sequencing

Axillary microbial genomic DNA was extracted from swab samples using the DNeasy PowerSoil Pro Kit (Qiagen, Germany) with a modified method to obtain additional genomic DNA. The preservative solution was transferred to a 1.5 mL microcentrifuge tube and centrifuged at 15,000*g* for 2 min. The pellet was suspended in a CD1 solution that was included in the extraction kit and was transferred to a PowerBead Pro tube with each swab head. After cell lysis of each sample, genomic DNA was extracted by following the manufacturer’s protocols. To prepare a skin microbiome library for sequencing, the 16S rRNA gene was amplified at V4 to V5 hypervariable region using a 515 F (5′-barcode-CGCTCTTCCGATCTGTGNCAGCMGCCGCGGTRA-3′) forward primer attached to 5′ Illumina adapter and 907 R (5′-barcode-GTGCTCTTCCGATCCGYCWATTYHTTTRAGTTT-3′) indexed reverse primer. Sequencing of all samples was performed with an Illumina MiSeq using a MiSeq Reagent Kit v3 (300 bp paired-end reads) (Illumina, Inc.).

### Sequencing data processing and network analysis

Quality check of the raw sequence data was performed using a Quantitative Insights into Microbial Ecology (QIIME) pipeline (version 1.9.1)^85^. The total reads were grouped into OTUs using the Greengenes database^86^ with a sequence identity threshold of 97%. All samples were rarefied to a depth of 2,720 reads. Furthermore, a prediction of the metabolic functions in the axillary microbiome was performed using the PICRUSt algorithm^87^. For a network analysis of the axillary microbiome, we used SPIEC-EASI^88^. OTUs < 0.01% were filtered for network analysis. We constructed the network of the axillary microbiome using the neighborhood algorithm and the Stability Approach to Regularization Selection method with a minimum lambda threshold of 0.1. This analysis was performed using the SPIEC-EASI R package (version 1.1.0). The network topological statistic coefficients were calculated using igraph R package (version 1.2.6). These features includes nodes (number of OTUs), edges (number of potential links between associated nodes), density (ratio of the number of nodes and the number of possible edges), degree (mean number of connected edges per node), transitivity (the probability that the adjacent nodes of a node are connected), and node closeness centrality (the number of steps required to access other nodes from one node).

### Bioconversion of *L. corniculatus* seeds using *L. acidophilus* KNU-02

*Lotus corniculatus* seeds were grinded and extracted with 70% ethanol at the ratio of 1:9. The filtered sample extract was dried using a SPD1010 SpeedVac System (Thermo Fisher Scientific, Waltham, MA, USA) at 45 °C for 2 h. A LAB isolate, *L. acidophilus* KNU-02, was cultivated in MRS (de Man, Rogosa, Sharpe) broth (Difco, Sparks, MD, USA) for 2 d at 37 °C in a shaking incubator at 180 rpm. The activated bacterial cells were obtained by centrifugation and washed with phosphate-buffered saline (pH 7.4). The bacterial cells were suspended (1.0 × 10^8^ cells/mL) in a phosphate-buffered saline mixed with 100 mg/mL of the *L. corniculatus* seed extract (100 mg/mL) in the ratio of 19:1 (v/v) and incubated for 4 d at 37 °C with shaking at 180 rpm. After centrifugation of the mixture, 1 volume of ethyl acetate with 0.1% acetic acid was added to the supernatant. The resulting solution was mixed thoroughly and placed for 5 min. The upper-layered solution was collected and dried using the SPD1010 SpeedVac System (Thermo Fisher Scientific, Waltham, MA, USA) at 45 °C for 2 h. The product was prepared with 3% 1,2-hexanediol, 0.16% tromethamine, 0.05% ethylhexylglycerin, 20% cabomer, and 0.1% extract powder (v/v).

### Compositional analysis

To investigate the compounds in the BLC and LC, a compositional analysis was performed by using GCxGC/TOF–MS. The compositional analysis was conducted using three replicates of each extract. Then, 100 mg of the dried extracts was transferred to a 2 mL centrifuge tube with 1 mL isopropanol–acetonitrile aqueous solution (3:2:2, v/v/v) added to the tube. The mixtures were vortexed for 10 min, followed by centrifugation at 8,000 g for 8 min. The supernatant was filtered with a 0.22 μm polyvinylidene fluoride filter, and 100 μL filtered supernatant was dried using a nitrogen evaporator. For the derivatization reaction, 100 μL methoxyamine hydrochloride in pyridine (20 mg/mL) was added to the dried extract and incubated at 30 °C for 90 min. Then, for silylation, 50 μL N-methyl-N-(trimethylsilyl) trifluoroacetamide (MSTFA) was added and incubated at 37 °C for 30 min. GCxGC-TOFMS, an Agilent 7890B Gas Chromatograph coupled with a LECO Pegasus 4D time-of-flight mass spectrometer (TOFMS) (Leco, St. Joseph, MI, USA), was used for the compositional analysis of the BLC. A column was used with an Rtx-5MS (Restek, Bellefonte, PA) with a length of 30 m, an internal diameter of 0.25 mm, and film thickness of 0.25 μm. The carrier gas was helium with a flow rate of 1.5 mL/min. A split ratio for the injector was set to 20:1, with a total injection volume of 1 μL. The front inlet, ion source, and transfer temperature were kept at 250 °C, 200 °C, and 260 °C, respectively. The oven temperature was maintained at 80 °C for 2 min, and the sample was injected. The temperature was increased at a rate of 15 °C/min to 200 °C. Then, the temperature was further increased by 5 °C/min to reach 300 °C and held for 3 min to complete the run. The MS was set at -70 eV with 1600 V of detector voltage. The mass scan range was m/z 35–650, and the acquisition rate was 15 Hz.

### Statistical analysis

All microbial analysis in this study was conducted within R software (version 4.0.2; http://www.r-project.org/)^89^. CAP allows a constrained ordination based on any distances or dissimilarity^90^. This method maximizes the separation of samples by continuous or factorial variables. CAP of the axillary microbiome was performed with an unweighted UniFrac distance matrix. We performed ANOVA to analyze the association between the microbiota and odor intensity using CAP analysis. This analysis was performed using the phyloseq (version 1.32.0) and vegan (version 2.5-6)^91,92^ R packages. A Wilcoxon matched-pairs test and a paired t-test were performed to compare 0 week with 1 week. The comparison of the baseline information for the BLC and placebo groups was performed using an unpaired t-test. A correlation analysis was performed using Spearman’s rank correlation coefficient with the Hmisc R package (version 4.4-1). A fold-change analysis of taxa and predicted genes was conducted using the DESeq2 R package (version 1.28.1). The *p* value in the fold-change analysis was calculated using multiple tests through the FDR (Benjamini-Hochberg). To adjust the effect of other taxa to one taxon, the multivariable regression model was used for investigating the association between taxa and odor intensity. This analysis was performed using MaAsLin2 R package (version 1.4.0). PLS-DA is a multiple linear regression technique to find the direction of maximum covariance between a data set and the class membership^93^. A VIP score is calculated as a weighted sum of the squared correlations between the PLS-DA components and the original variable. Therefore, PLS-DA was performed using a MetaboAnalyst for a compositional analysis, and the comparison of discriminant feature between two extracts was conducted using two-tailed student’s t-test^94^.

## Supplementary Information

Supplementary Information.
