# Supplementary material for: Effect of a bioconverted product of Lotus corniculatus seed on the axillary microbiome and body odor
Source: Sci Rep. 2021 May 12;11:10138. doi: 10.1038/s41598-021-89606-5 (PMC8115508; doi:10.1038/s41598-021-89606-5)
Supplement: Supplementary file 1 — Supplementary Information. [file 41598_2021_89606_MOESM1_ESM.pdf]

Supplementary information

Effect of a Bioconverted Product of *Lotus corniculatus* Seed on the Axillary Microbiome and Body Odor

Min-Ji Kim[1], Setu Bazie Tagele[1], HyungWoo Jo[2], Min-Chul Kim[1], YeonGyun Jung[1], Yeong-Jun Park[1], Jai-Hyun So[3], Hae Jin Kim[4], Ho Jin Kim[4], Dong-Geol Lee[2], Seunghyun Kang[2], and Jae-Ho Shin[1*]

[1]Department of Applied Biosciences, Kyungpook National University, Daegu, 41566, Republic of Korea

[2]R&I Center, COSMAX BTI, Seongnam-si, 13486, Republic of Korea

[3]National Development Institute of Korean Medicine, 94, Hwarang-ro, Gyeongsan, Gyeongsangbuk-do, 38540, Republic of Korea

[4]Experiment Research Institute, National Agricultural Products Quality Management Service, Gyeongsangbuk-do 39660, Republic of Korea

[*]Corresponding Author's information

Kyungpook National University, Daegu 41566, Republic of Korea
(Tel): +82-53-950-5716
(Fax): +82-53-953-7233

(E-mail): jhshin@knu.ac.kr

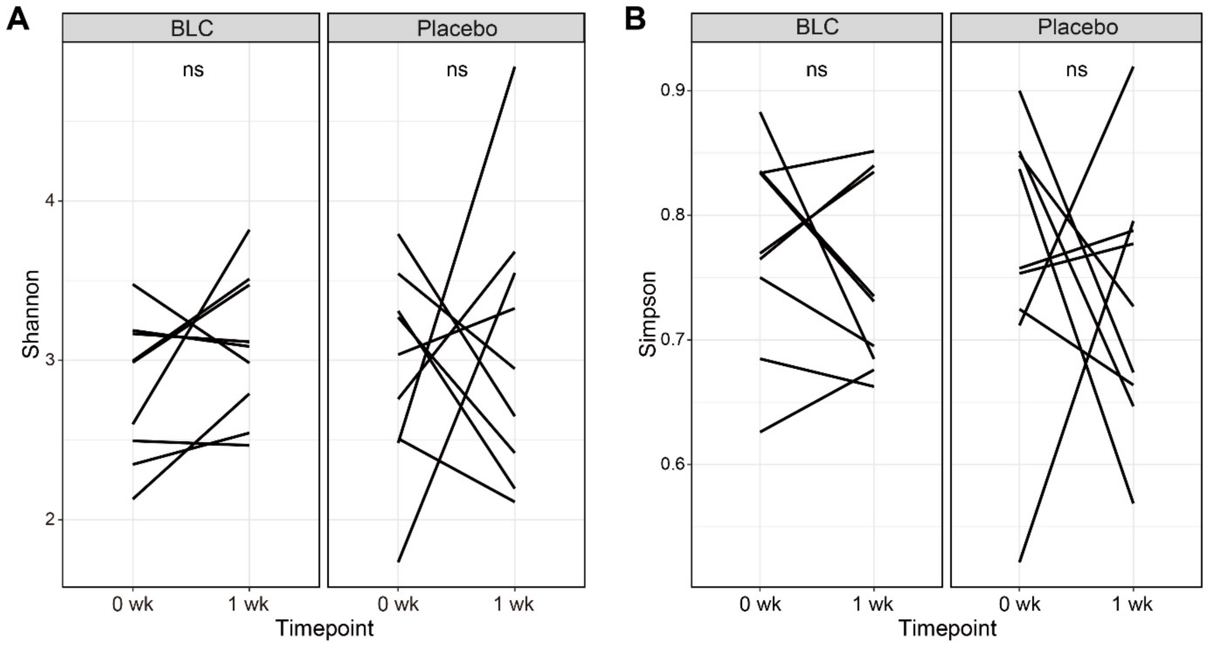

Figure S1. Alpha diversity of the axillary microbiome in each group. **(A)** Shannon and **(B)** Simpson's index in bioconverted product (BLC) and placebo group. Wilcoxon matched-pairs test was conducted to compare between 0 wk and 1 wk.

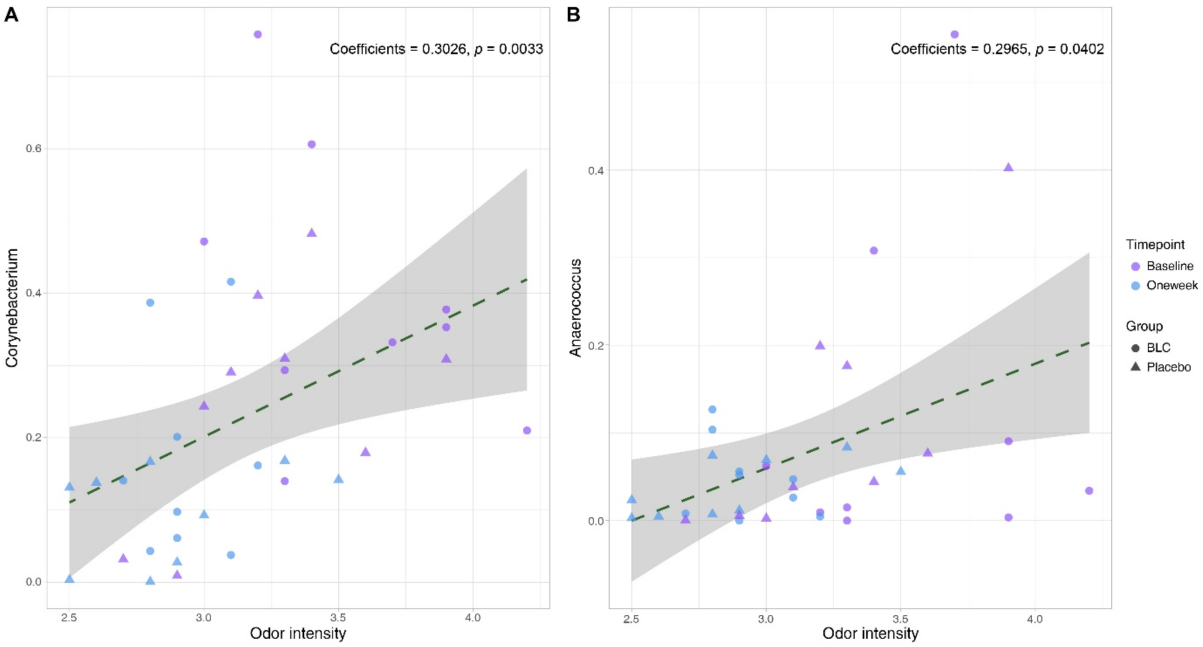

Figure S2. Relative abundance of (A) *Corynebacterium* and (B) *Anaerococcus* with linear regression fit to odor intensity.

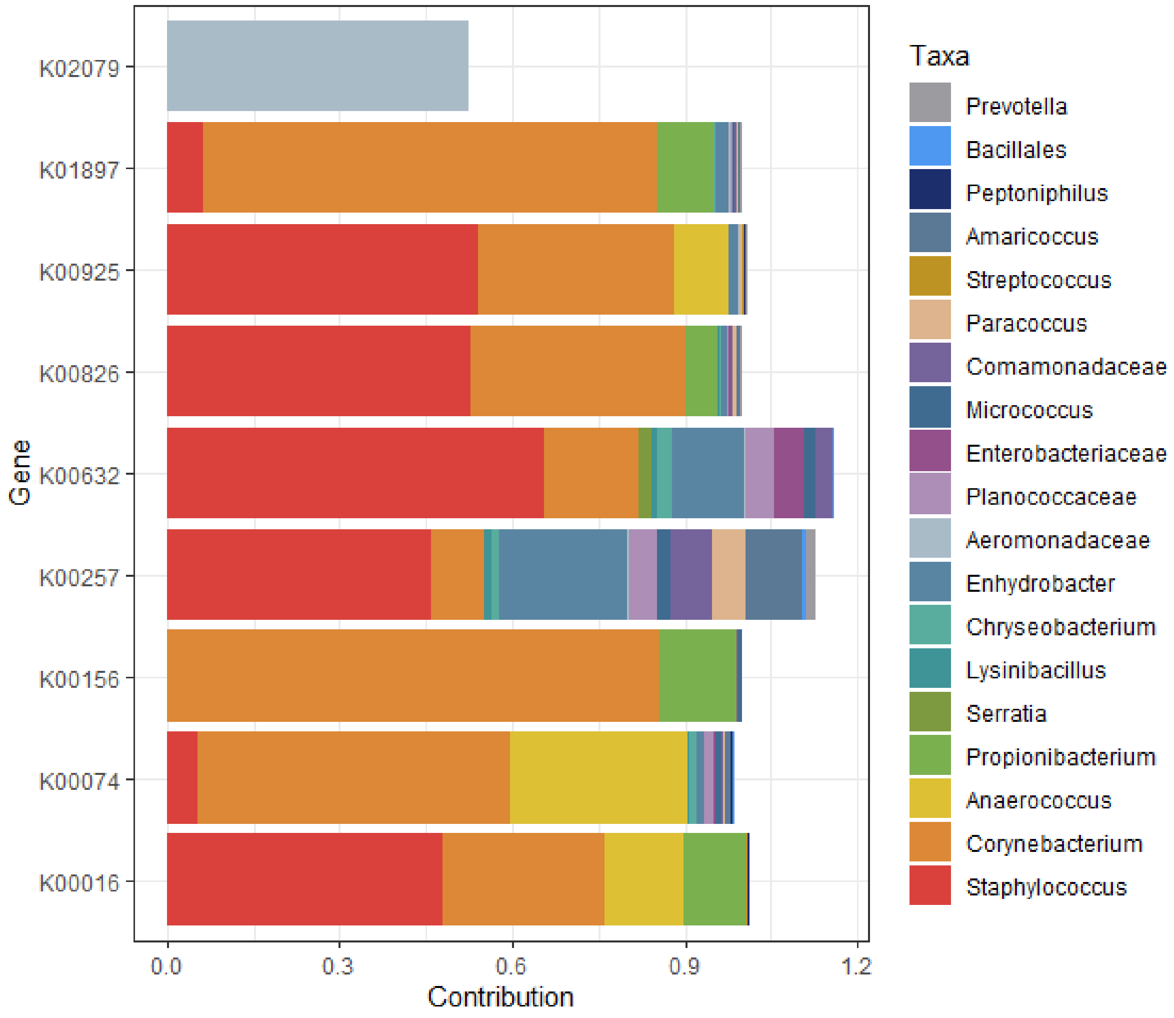

Figure S3. The contribution of taxa to the odor-related genes. Color indicates abundant taxa in axillary microbiome. Contribution was calculated as contribution of each taxon in the each sample to odor-related genes.

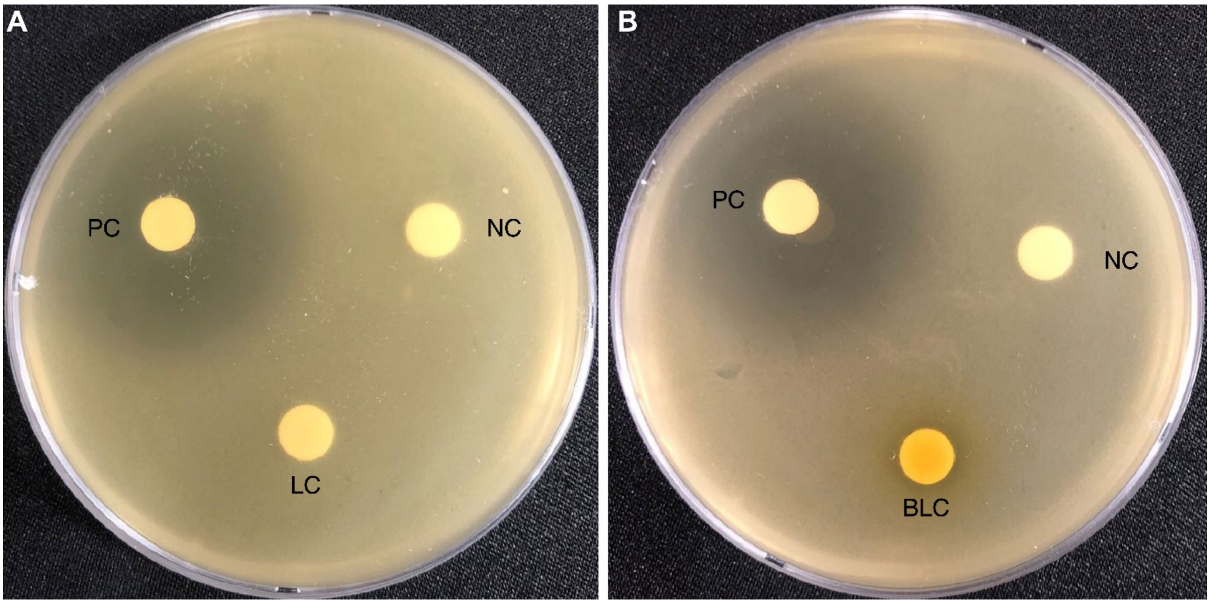

Figure S4. Antibacterial activity of (A) original seed extract (LC) and (B) bioconverted product of *Lotus corniculatus* seed (BLC) against *Staphylococcus aureus* KCCM 40510. PC, positive control (2 mg/mL chloramphenicol); NC, negative control (methanol); LC, original seed extract; BLC, bioconverted product of *Lotus corniculatus* seed.

Table S1. The list of major compounds (>1%) identified in seed extract by GCxGC/TOF-MS analysis

Compound	R.T. (s)	MW (g/mol)	Formula	Area (%)
Methyl galactoside (1R,2R,3S,4S,5R)-, 4TMS derivative	1718.1	482.90	$C_{19}H_{46}O_6Si_4$	15.45
2-(Dimethylamino)ethanol, TMS derivative	244.73	161.32	$C_7H_{19}NOSi$	10.04
6,7-Dihydroxycoumarin-á-D-glucopyranoside, PENTA-TMS	1470.8	700.00	$C_{30}H_{56}O_9Si_5$	5.000
Propylene glycol, 2TMS derivative	304.70	220.46	$C_9H_{24}O_2Si_2$	4.265
Propanoic acid, 3-[(trimethylsilyl)oxy]-, trimethylsilyl ester (CAS)	400.76	234.44	$C_9H_{22}O_3Si_2$	3.245
Ethyl à-D-glucopyranoside, 4TMS derivative	795.25	496.90	$C_{20}H_{48}O_6Si_4$	2.721
L-Proline, 5-oxo-1-(trimethylsilyl)-, trimethylsilyl ester (CAS)	620.32	273.48	$C_{11}H_{23}NO_3Si_2$	2.716
Silanol, trimethyl-, phosphate (3:1) (CAS)	483.46	314.54	$C_9H_{27}O_4PSi_3$	2.432
D-Pinitol, pentakis(trimethylsilyl) ether	803.79	555.09	$C_{22}H_{54}O_6Si_5$	2.363
Glycerol, 3TMS derivative	481.20	308.64	$C_{12}H_{32}O_3Si_3$	2.067
Homoserine, 3TMS derivative	578.49	335.66	$C_{13}H_{33}NO_3Si_3$	1.982
4-Aminobutanoic acid, 3TMS derivative	620.96	319.66	$C_{13}H_{33}NO_2Si_3$	1.860
D-Psicose, pentakis(trimethylsilyl) ether, methyloxime (anti)	828.64	570.10	$C_{22}H_{55}NO_6Si_5$	1.503
Cyclopentasiloxane, decamethyl- (CAS)	399.73	370.77	$C_{10}H_{30}O_5Si_5$	1.475
D-GLUCURONIC ACID PK B 5TMS	1233.8	555.05	$C_{21}H_{50}O_7Si_5$	1.212
Ethanolamine, 3TMS derivative	478.74	277.63	$C_{11}H_{31}NOSi_3$	1.127
L-Tyrosine, N,O-bis(trimethylsilyl)-, trimethylsilyl ester (CAS)	870.52	397.70	$C_{18}H_{35}NO_3Si_3$	1.104
L-Alanine, N-(trimethylsilyl)-, trimethylsilyl ester (CAS)	374.89	233.46	$C_9H_{23}NO_2Si_2$	1.058
L-Aspartic acid, 3TMS derivative	614.58	349.65	$C_{13}H_{31}NO_4Si_3$	1.018

Table S2. The list of compounds significantly different between seed extract and bioconverted product (VIP >0.7)

Compound	VIP score	*p*-value
6,7-DIHYDROXYCOUMARIN--D-GLUCOPYRANOSIDE, PENTA-TMS	15.44	0.000003
2-Dimethylaminoethanol, TMS derivative	3.254	0.000521
Methyl galactoside 1R,2R,3S,4S,5R-, 4TMS derivative	2.424	0.000004
Propylene glycol, 2TMS derivative	1.508	0.000383
Propanoic acid, 3-trimethylsilyloxy-, trimethylsilyl ester CAS	1.153	0.000163
Ethyl -D-glucopyranoside, 4TMS derivative	1.072	0.000012
L-Proline, 5-oxo-1-trimethylsilyl-, trimethylsilyl ester CAS	1.063	0.000008
Homoserine, 3TMS derivative	0.796	0.000004
Glycerol, 3TMS derivative	0.787	0.000060
D-Pinitol, pentakistrimethylsilyl ether.1	0.775	0.000002
Benzoic acid, 3,4-bistrimethylsilyloxy-, trimethylsilyl ester CAS	0.760	0.029903
4-Aminobutanoic acid, 3TMS derivative	0.742	0.000004

Table S3. Baseline information of the study participants

	BLC	Placebo	*P* value
Sample size (n)	9	9	
Age (years)	48.7 ± 8.20	43.6 ± 7.93	0.2269
Axillary odor intensity	3.50 ± 0.40	3.20 ± 0.37	0.1038

Table S4. Network topological statistic coefficients in each group

Group	Time point (wk)	Nodes	Edges	Density	Transitivity	Degree	Node closeness centrality
BLC	0	203	360	0.018	0.510	3.547	0.00011
	1	322	1085	0.021	0.581	6.739	0.00035
Placebo	0	228	588	0.023	0.546	5.158	0.00012
	1	362	1366	0.021	0.673	7.547	0.00014

Table S5. Relative abundance of odor-related genes in each group

Genes	BLC (n = 9)		Placebo (n = 9)	
	0 wk	1 wk	0 wk	1 wk
L-lactate dehydrogenase	0.09293 ± 0.01777	0.07950 ± 0.01303	0.09738 ± 0.20630	0.09344 ± 0.05328
3-Hydroxybutyryl-CoA dehydrogenase	0.04186 ± 0.03336	0.02454 ± 0.01133	0.02885 ± 0.02557	0.01912 ± 0.01633
Pyruvate dehydrogenase	0.03493 ± 0.01751	0.01814 ± 0.01241	0.02628 ± 0.01175	0.02095 ± 0.02017
Acyl-ACP dehydrogenase	0.00871 ± 0.00646	0.02463 ± 0.01632	0.00960 ± 0.00531	0.02622 ± 0.03181
Acetyl-CoA acyltransferase	0.00484 ± 0.00212	0.01347 ± 0.00774	0.00636 ± 0.00235	0.01499 ± 0.01651
BCAA aminotransferase	0.07203 ± 0.00926	0.07416 ± 0.00356	0.07132 ± 0.00599	0.07390 ± 0.00310
Acetate kinase	0.07760 ± 0.00552	0.06737 ± 0.00490	0.07069 ± 0.01114	0.05805 ± 0.02164
Long-chain acyl-CoA synthetase	0.11903 ± 0.05473	0.07191 ± 0.03604	0.08785 ± 0.03620	0.07291 ± 0.03665
N^{α}-acylglutamine aminoacylase	0.00002 ± 0.00006	0.00033 ± 0.00039	0.00003 ± 0.00009	0.00030 ± 0.00062
Sum of odor-related genes	0.45194 ± 0.10797	0.37403 ± 0.06904	0.39897 ± 0.07546	0.37986 ± 0.08233
